# A retrospective controlled study protocol of transforaminal lumbar interbody fusion compared with posterior lumbar interbody fusion for spondylolisthesis

**DOI:** 10.1097/MD.0000000000022878

**Published:** 2020-10-30

**Authors:** Ping Yi, Xiangsheng Tang, Feng Yang, Mingsheng Tan

**Affiliations:** Department of Orthopedic Surgery, China-Japan Friendship Hospital, Ministry of Health, Beijing, China.

**Keywords:** posterior lumbar interbody fusion, spondylolismia, study protocol, transforaminal lumbar interbody fusion

## Abstract

**Background:**

In the current literature, for adult lumbar spondylolisthesis, the direct comparison of clinical outcomes and perioperative complications between transforaminal lumbar interbody fusion (TLIF) and posterior lumbar interbody fusion (PLIF) is limited. Whether the therapeutic effect of TLIF is better than that of PLIF is still controversial. In this retrospective controlled study, our aim was to compare their clinical outcomes and radiological results of the above two stabilization approaches after 1-year follow-up period.

**Methods:**

This investigation was approved via the Institutional Review committee of China-Japan friendship hospital. This was a retrospective single-center analysis of subjects. We reviewed the patients with spondylolisthesis treated with TLIF or PLIF between July 2016 and February 2019 in our hospital. Patients with these conditions will be included: with the radiological evidence of degenerative lumbar spondylolismia with leg pain and/or low back pain, or the neurogenic claudication after failure of conventional conservative treatment for more than 6 months. The patients who received 3 levels or more intervertebral fusion levels were excluded. Patients without a completed medical history were excluded. Patients who had a history of lumbar spine surgery were also excluded. Clinical outcomes in our follow-up included functional outcomes, complications, and radiographic such as spondylolisthesis degree. The radiographs were obtained at 1, 3, 6, and 12 months during the outpatient follow-up.

**Results:**

This protocol will provide a solid theoretical basis for exploring which technique is better in treatment of spondylolisthesis.

**Trial registration:**

This study protocol was registered in Research Registry (number: researchregistry6032).

## 1 Introduction

Spinal stability refers to the ability of the vertebrae to limit their relative displacements and maintain their relationships during loads and physiological postures.^[1]^ Degenerative lumbar spondylolisthesis (DLS), also known as lumbar arthritis spondylolisthesis, is a kind of degenerative spine disease that general leads to leg and low back pain associated with spinal stenosis. In general population, the prevalence of DLS ranged from 4.1% to 11.1%, which was defined as the spondylitislisthesis with no interarticular defect.^[2–5]^

Surgical approaches are utilized to treat degenerative lesions that develop due to minor trauma and age and do not respond to drug therapies. Two techniques utilized in treating the spondylolisthesis are transforaminal lumbar interbody fusion (TLIF) and posterior lumbar interbody fusion (PLIF).^[6–9]^ PLIF includes the 2 cages insertion through the bilateral approach, which has become a standard surgical technique for the lumbar spondylolisthesis.^[10,11]^ The posterior approach can achieve the stable 3-column fixation, which can fuse 360°. Nevertheless, during the operation of intervertebral disc space, the thecal sac needs to be retracted, which increases the risk of nerve root injury and durotomy.^[12]^ The TLIF procedure was designed to decrease the risks related to the PLIF procedure.^[13,14]^ For the TLIF, unilateral transforaminal path was utilized to intervertebral space, the unilateral facet arthroplasty was performed, and a cage was inserted. Several former researches have assessed clinical outcomes after PLIF and TLIF, and then reporting that the TLIF is safer and less invasive than PLIF. Furthermore, some surgical complications related to the unilaterally cages-insertion utilized in the TLIF have also been reported.^[15–18]^

In the current literature, for adult lumbar spondylolisthesis, the direct comparison of clinical outcomes and perioperative complications between TLIF and PLIF is limited. Whether the therapeutic effect of TLIF is better than that of PLIF is still controversial. In this retrospective controlled study, our aim was to compare their clinical outcomes and radiological results of the above 2 stabilization approaches after 1-year follow-up period.

## 2 Materials and methods

### 2.1 Ethical approval

This investigation was approved via the Institutional Review committee of China-Japan friendship hospital (number: K-N562). On the basis of institutional guidelines, the informed consent could be acquired. This current investigation was registered with the Research Registry (number: researchregistry6032).

### 2.2 Study design and population

This was a retrospective single-center analysis of subjects. We reviewed the patients with spondylolisthesis treated with TLIF or PLIF between July 2016 and February 2019 in our hospital.

Patients with these conditions will be included: with the radiological evidence of degenerative lumbar spondylolismia with leg pain and/or low back pain, or the neurogenic claudication after failure of conventional conservative treatment for more than six months. The patients who received 3 levels or more intervertebral fusion levels were excluded. Patients without a completed medical history were excluded. Patients who had a history of lumbar spine surgery were also excluded.

### 2.3 Operative procedure

The details of operation and the latent complications are recorded in patient records and then explained to each patient. After proper explanation of the management options and surgical technique, an informed written consent was taken. The patients were operated under general anesthesia. The patient was placed on operating table with the semi flexible prone position.

For the TLIF group, the patient was inserted with pedicle screws via the midline approach. The hemilaminectomy and unilateral medial facet joint resection were implemented with the TLIF. After the intervertebral disc space was cleaned, polyetheretherketone cage was filled by using the autogenous bone. After the cage was placed, the intervertebral disc space was compressed to produce lordosis. Facet joint, spinous process, and autogenous lamina were utilized as the bone graft materials and they placed on the transverse process of vertebrae fused.

For the PLIF group, PLIF approach was utilized for the medial facet joint resection and total laminectomy. After the pedicle screw was inserted, through bilateral approach, the discectomy was carried out. After the autologous bone grafting was implemented in the area of anterior endplate, 2 cages filled with the autologous bone grafting were inserted into the endplate on both sides. Peek cages filled by using the autologous bone graft on both sides were utilized for the intervertebral fusion. If necessary, an ultimate fluoroscopy is carried out to confirm the cage placement and pedicle screw fixation.

### 2.4 Outcome evaluation

The perioperative factors associated with the surgical procedure (for instance, the demographics of patients, duration of surgery, hospital stay as well as surgical blood loss) were recorded. Clinical outcomes in our follow-up included functional outcomes, complications, and radiographic such as spondylolisthesis degree. The radiographs were obtained at 1, 3, 6, and 12 months during the outpatient follow-up.

Functional outcomes in our follow-up were assessed using Oswestry Disability Index (ODI) and the scores of Japanese Orthopedic Association (JOA). The ODI is an existing tool, which was originally designed to detect the disability caused by the lowback pain, and it involves 10 questions designed to evaluate the limitations in a variety of areas of activities of daily living, with an emphasis on lower-extremity activities. The score of JOA was conducted for the assessment of the patients with low back pain. The score of JOA evaluate pain and functionality, including 4 sections (14 items). The total score of the questionnaire is between -6 and +29, the higher the score, the better the situation.

### 2.5 Statistical analysis

The statistical analysis could be carried out with the software of Statistical Package for Social Sciences version 20.0 (IBM Corporation, Armonk, NY). The nonparametric tests and parametric tests are applied appropriately to evaluated the significant differences in continuous variables between the groups. The linear variables were compared between the groups with the Student *t* test. For the dichotomous variables, it can be evaluated with the Chi-square test.

## 3 Discussion

The optimal surgical approach to the treatment of adult lumbar spondylolisthesis has not been determined yet. Currently, PLIF and TLIF are both utilized to treat the lumbar spondylolisthesis, although they have been improved with the introduction and development of novel implants. In this retrospective controlled study, our aim was to compare their clinical outcomes and radiological results of the above 2 stabilization approaches after 1-year follow-up period. The limitations of this current investigation contained the inherent limitations in any existing retrospective cohort research, involving the possibility of observation bias and selection.

## Author contributions

**Conceptualization:** Ping Yi, Feng Yang, Xiangsheng Tang.

**Data curation:** Ping Yi, Xiangsheng Tang.

**Formal analysis:** Ping Yi, Xiangsheng Tang, Feng Yang.

**Funding acquisition:** Mingsheng Tan.

**Investigation:** Ping Yi, Xiangsheng Tang.

**Methodology:** Feng Yang.

**Project administration:** Mingsheng Tan.

**Software:** Feng Yang, Xiangsheng Tang, Ping Yi.

**Supervision:** Mingsheng Tan.

**Validation:** Xiangsheng Tang.

**Visualization:** Feng Yang, Mingsheng Tan.

**Writing – original draft:** Ping Yi.

**Writing – review & editing:** Mingsheng Tan.
